# Pulsed Electromagnetic Therapy: Literature Review and Current Update

**DOI:** 10.1590/0103-6440202406109

**Published:** 2024-10-25

**Authors:** Yaniv Mayer, Jamil Awad Shibli, Haia Abu Saada, Marcelo Melo, Eran Gabay, Shlomo Barak, Ofir Ginesin

**Affiliations:** 1 Department of Periodontology, school of graduate dentistry, Rambam Health Care Campus (RHCC), Haifa, Israel.; 2 Faculty of Medicine, Technion - Israel Institute of Technology, Haifa, Israel.; 3 Department of Periodontology and Oral Implantology, University of Guarulhos, Guarulhos, Brazil.

**Keywords:** Pulsed Electromagnetic Fields (PEMFs), Therapeutic Applications, Tissue Healing, Dental Implantology, Biological Processes

## Abstract

This manuscript provides a comprehensive review of Pulsed Electromagnetic Fields (PEMFs), highlighting their therapeutic potential and historical evolution. PEMFs, recognized for their non-invasive and safe therapeutic benefits, interact with biological systems to influence processes such as DNA synthesis, gene expression, and cell migration. Clinically, PEMFs are applied in diverse treatments, including pain relief, inflammation management, and enhancing bone and wound healing. The manuscript delves into the historical development of PEMF technology, tracing its origins to the 19th century and exploring significant advancements, such as the discovery of the piezoelectric effect in bones. It presents detailed in-vitro and in-vivo studies demonstrating PEMFs' impact on cellular activities and their modulation of key biological pathways. Additionally, the review emphasizes PEMFs' applications in general medicine and dentistry, showcasing their role in promoting tissue healing, osseointegration in dental implants, and antimicrobial effects. The introduction of the Miniaturized Electromagnetic Device (MED) in dental implantology marks a significant advancement, enhancing implant stability and reducing inflammatory responses. Overall, the manuscript underscores PEMFs' promising applications in advancing patient care and treatment methodologies across medical and dental fields.

## Resumo

Este manuscrito oferece uma revisão abrangente dos Campos Eletromagnéticos Pulsados (PEMFs), destacando seu potencial terapêutico e evolução histórica. Os PEMFs, reconhecidos por seus benefícios terapêuticos não invasivos e seguros, interagem com sistemas biológicos para influenciar processos como síntese de DNA, expressão gênica e migração celular. Clinicamente, os PEMFs são aplicados em diversos tratamentos, incluindo alívio da dor, manejo da inflamação e melhoria na cicatrização óssea e de feridas. O manuscrito explora o desenvolvimento histórico da tecnologia PEMF, rastreando suas origens ao século XIX e explorando avanços significativos, como a descoberta do efeito piezoelétrico nos ossos. Apresenta estudos detalhados in vitro e in vivo demonstrando o impacto dos PEMFs nas atividades celulares e sua modulação de vias biológicas chave. Além disso, a revisão enfatiza as aplicações dos PEMFs na medicina geral e odontologia, mostrando seu papel na promoção da cicatrização de tecidos, osseointegração em implantes dentários e efeitos antimicrobianos. A introdução do Dispositivo Eletromagnético Miniaturizado (MED) na implantologia dentária marca um avanço significativo, melhorando a estabilidade dos implantes e reduzindo respostas inflamatórias. No geral, o manuscrito destaca as aplicações promissoras dos PEMFs no avanço do cuidado ao paciente e das metodologias de tratamento nos campos médico e odontológico.

**Figure:**
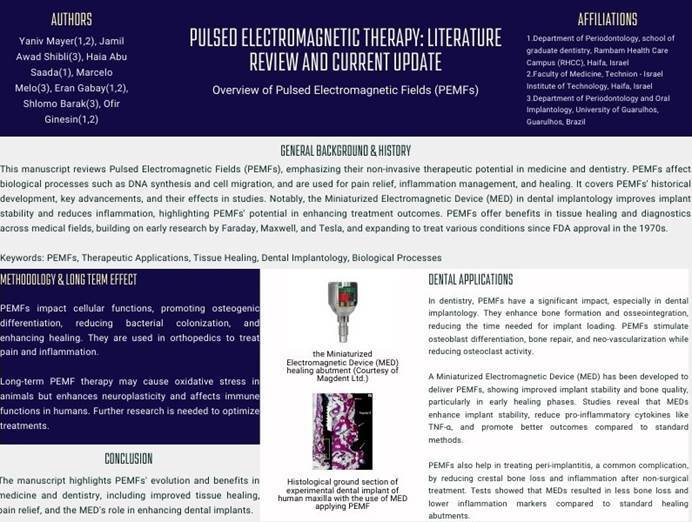

## Introduction

Pulsed Electromagnetic Fields (PEMFs) encompass both magnetic and electric fields that interact with one another^1^. This technology is recognized for its non-invasive and safe therapeutic benefits, Additionally, it plays a role in diagnosis, providing accurate assessments for various medical conditions^2^. PEMFs are distinguished by their ability to fully penetrate tissues, facilitating the detection of a range of pathologies in both humans and animals^1^.

Research has demonstrated that PEMFs exert a significant impact on numerous biological processes, including DNA synthesis, gene expression, and cell migration^3^. This biological activity is partly facilitated through the synthesis of cytokines, which play a crucial role in maintaining the body's homeostatic balance. Clinically, PEMFs have been applied in various treatments, offering relief from post-operative pain, managing inflammation-especially in cases of osteoarthritis-and aiding in bone and wound healing. The application of PEMFs in clinical settings is on the rise, with its utility expanding across a broad spectrum of medical indications^2^.

This review delves into the historical progression of PEMF technology, presents detailed in-vitro and in-vivo studies, and specifically emphasizes its applications in medical and dental treatments through PEMFs therapy.

## History

Electromagnetic field therapy, commonly known as PEMFs therapy, traces its origins back to the early 19th century following the groundbreaking work on electromagnetism by luminaries like Michael Faraday and James Clerk Maxwell. This period marked the beginning of a journey into understanding the therapeutic possibilities of electromagnetism. By the late 19th and early 20th centuries, figures such as Nikola Tesla began to explore the health benefits of electromagnetic fields, promoting the therapeutic use of high-frequency electromagnetic fields.

The discovery of the piezoelectric effect in bones by Yasuda in 1957 marked a pivotal moment in the mid-20th century, showcasing the potential of electromagnetic fields in promoting bone healing^4^. This discovery led to the creation of PEMFs therapy devices, initially designed to address bone fractures and non-unions resistant to conventional treatments.

As the years progressed, the application of electromagnetic field therapy broadened to encompass a wider range of conditions, including pain management, inflammation reduction, among others^2^. The FDA's endorsement of PEMFs devices for specific medical uses, such as bone healing in the 1970s, represented a crucial endorsement for the integration of electromagnetic field therapy into mainstream medical practices. The evolution of PEMFs technology continued with the introduction of new-generation devices capable of treating soft tissues in addition to bones.

## In-Vitro studies

Research has demonstrated the capacity of Pulsed Electromagnetic Fields (PEMFs) to significantly influence cellular activities such as the cell cycle, apoptosis, proliferation, and differentiation^2^ ^,^ ^3^. These effects are primarily attributed to the forced vibration of free ions on cell membrane surfaces, initiated by an external oscillating field. Such actions can lead to irregular gating of ion channels, potentially disrupting the equilibrium of transmembrane proteins and, as a result, cell functionality. PEMFs are believed to influence the entire signal transduction pathway, altering cellular behaviors and affecting both cell surface receptor expression and downstream signal transduction pathways^3^ ^,^ ^5^. This modulation can restore homeostatic functions including cell differentiation, viability, and proliferation, as well as interactions with the extracellular matrix and neighboring cells^5^ ^,^ ^6^. Furthermore, PEMFs enhance neurogenic and osteogenic differentiation of mesenchymal stem cells due to their ability to penetrate cells and alter the electric field within, impacting biological processes through modulation of Ca2+ efflux and signaling pathways like nitric oxide, growth factor secretion, and MAPK/ERK^3^.

PEMFs influence apoptosis by modulating several keys signaling pathways. For example, they activate the mitogen-activated protein kinase (MAPK) pathway, which includes ERK, JNK, and p38 MAPKs, leading to both pro-apoptotic and anti-apoptotic responses depending on the cell type and context. PEMFs can also inhibit the activation of NF-κB, a transcription factor involved in the regulation of apoptosis, thereby reducing the expression of pro-apoptotic genes.

In terms of cellular proliferation, PEMFs enhance this process through mechanisms such as the cAMP/PKA pathway. Exposure to PEMFs increases the levels of cyclic adenosine monophosphate (cAMP), which activates protein kinase A (PKA) and promotes cell proliferation. Additionally, PEMFs activate the Wnt/β-catenin signaling pathway, crucial for regulating cell proliferation and differentiation. This activation leads to the accumulation of β-catenin in the cytoplasm and its translocation to the nucleus, where it promotes the expression of proliferation-related genes.

PEMFs also facilitate cellular differentiation through several pathways. They stimulate the transforming growth factor-beta (TGF-β) and bone morphogenetic protein (BMP) pathways, vital for the differentiation of various cell types, including osteoblasts and chondrocytes. The mammalian target of rapamycin (mTOR) pathway is another target of PEMFs, promoting cell growth and differentiation by increasing protein synthesis. Insulin-like growth factors (IGFs) are upregulated by PEMFs, contributing to the differentiation of mesenchymal stem cells into osteoblasts and enhancing bone formation and repair.

PEMFs modulate these signaling pathways through various mechanisms. They affect ligand binding and distribution as well as the activity of several membrane receptors, including parathyroid hormone (PTH), insulin, IL-2, IGF-2, LDL, and calcitonin receptors. Moreover, PEMFs stimulate the synthesis of growth factors such as IGF, BMP, TGF-β, and PGE2, which are crucial for the regulation of cell proliferation, differentiation, and extracellular matrix (ECM) synthesis.

Study suggests that PEMFs also has an extensive effect of bone formation by interaction between Wet ligands with Bone Morphogenetic Proteins (BMPs) which is considered as complicated pathway in which these two signals interact depending on the developmental stage^4^ ^).^

Sensitivity to PEMFs varies among cell types, indicating a selective response to treatment^2^. This variability in sensitivity is influenced by differences in signal transduction pathways and cellular functions, as well as factors like frequency, intensity, and duration of PEMF exposure. For instance, PEMFs has shown promising therapeutic effects by enhancing Ca2+ signaling, which is crucial for processes related to apoptosis, inflammation, and metabolism^2^. It has been observed that PEMFs treatment accelerates the differentiation of bone marrow stromal cells and promotes the proliferation and differentiation of osteoblast cells through low frequency pulsed electromagnetic therapy, showcasing its potential in therapeutic fields by modulating key cellular processes^7^. On different titanium surfaces, osteoblast proliferation was increased and directed peripendicular to PEMFs field^8^. Osteoblast also showed higher number of microfilaments and higher expression of osteogenesis-related genes^8^.

**Figure 1:**
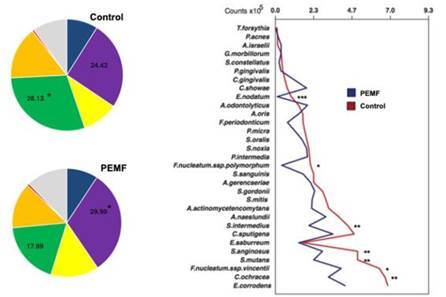
A) Pie charts of the mean proportions of each microbial complex in the In vitro multispecies biofilm. Different colors represent the microbial complexes described by Socransky *et al.* (1998). The grey color (‘Others’) represents species that did not fall into any complex, and Actinomyces spp. are represented in blue. Significance of differences in mean proportions between groups were tested using the Mann-Whitney U-test (*p>0.05); B) Mean bacterial count (x10^5^) of the bacterial species formed on titanium surfaces of Control group, healing cap without electromagnetic healing cap and Test group, healing cap with electromagnetic healing cap. Data were analyzed using the Mann-Whitney test (* p<0.05; ** p<0.01, *p<0.001). Reproduced with permission (Faveri et al. Biofouling 2021 License Number 5733760205595).

Based on in vitro studies PEMFs also affect bacterial colonization. Faveri et al examined PEMFs bacterial influence using a polymicrobial periodontal subgingival biofilm model^9^. They found that more than 25% of the bacterial species differed significantly in implants connected to MED compared to standard healing abutment (Control group) and total bacterial load was lower in MED group^9^. Initial biofilm colonization of important species including, *S. anginosus, F. nucleatum, S. intermedius, F. nucleatum* were found in higher levels in the control group compared with the MED group^9^. Worth mentioning that also the lower level of *s. mutans* were found in the MED group compared with the control group^9^ (Figure 1).

## In-Vivo studies

In-vivo studies suggest additional mechanisms. PEMFs applied for 30 min in rats resulted in decreased tissue hypoxia, attenuated neuronal necrosis through arteriolar dilation, enhanced capillary blood flow, reduced microvascular shunt/capillary ratio, and potentially increased blood-brain barrier permeability^10^. Another study in diabatic rats focused on the effect of PEMFs on myofibroblast population and results showed a rise of collagen fiber production leading to early wound healing^11^ **.**

PEMFs was also showed promising results treating stroke^12^. After injection of bone marrow mesenchymal stem cells treated with PEMFs, inflammation resolution in the ischemic area was found^12^.

In dental implantology, PEMFs was used on titanium implants placed in rabbit tibia^13^. Results showed elevation of osteogenesis-related genes including Runx2, OSX, COL-1 and Wnt/β-catenin as well as induction of mature cytoskeleton. μCT and histomorphometry results were consistent with the gene-level findings with better performance in the PEMFs group^13^.

## Application in general medicine

Modern medicine has various successful applications using magnetic and electromagnetic stimulation which known as a safe therapy. PEMFs exhibit biomodulatory effects on various cellular and molecular signaling pathways, offering potential therapeutic implications for a range of pathological conditions.

PEMFs stimulate vasodilation which enhance the management of post-operative pain and edema, chronic wound therapy which can moderate angiogenesis^14^, PEMFs could promote the production of anti-inflammatory mediators, potentially leading to reduced inflammation, rise angiogenic factor of TCs culture models, and tendon-specific gene expression^15^. It can also affect cell membrane response through Adenosine and its receptors in bone homeostasis^16^, inhibiting osteoclast differentiation and increasing new bone formation raw in bone defects^17^. These properties allow PEMFs to be widely used in orthopedic treatments with remarkable success and improving therapeutic outcomes mainly in osteoarthritis, osteoporosis, osteonecrosis, tendon disorders^18^ and skeletal pathology^19^.

In addition, PEMFs devices has been used to various medical applications including pain syndromes^20^. For example, interstitial cystitis/bladder pain syndrome (IC/BPS)^21^. Several treatment protocols including full body or targeted treatment were suggested to patients dealing with IC/BPS both treatment modalities with promising results^21^.

PEMFs was also used in Parkinson, multiple sclerosis, pain relief and delayed fracture^22^, through selected weak PEMFs that helped initiating healing process^22^. PEMFs can be useful also in combination with other technologies, for example, combination of PEMFs and ozone gas improve ovarian function in women undergoes in-vitro fertilization^23^.

Studies emphasize the role of signal characteristics such as amplitude and frequency in determining biological responses to PEMFs, suggesting the necessity of optimizing these parameters for therapeutic applications^2^. Optimizing PEMF parameters involves careful consideration of amplitude, frequency, pulse duration, and treatment duration. For instance, low intensities (1-10 Gauss) are effective for chronic pain and inflammation, while medium intensities (15-30 Gauss) are used for bone healing. Frequencies like 2 Hz can improve sleep, 50 Hz can reduce pain and inflammation, and 200-300 Hz can aid in bone growth. Short pulse durations are ideal for acute pain relief, and longer pulses benefit chronic conditions.

## PEMF compared to other modalities

Other modalities of therapies, such as low-level laser therapy (LLLT) and ultrasound, exists in the field, and comparing them with pulsed electromagnetic fields (PEMFs) demonstrated that PEMFs offer distinct advantages in various medical and dental applications. PEMF therapy has shown significant effectiveness in reducing pain and promoting healing in conditions such as diabetic foot ulcers and myofascial pain compared to laser therapy. For instance, PEMFs significantly decreased pain and improved wound closure rates in diabetic foot ulcers, showcasing an enhanced ability to reduce inflammation and promote angiogenesis compared to laser therapy^24^. Additionally, PEMFs exhibited a dual effect on muscles, including heating and molecular resonance, which contributed to muscle lengthening and ischemia reduction, leading to greater pain relief in myofascial pain treatment than laser therapy^25^.

However, laser therapy also demonstrated substantial benefits, particularly in wound regeneration, by stimulating cellular processes such as fibroblast proliferation and collagen synthesis. Studies have indicated that laser therapy significantly enhances wound healing, reduces bacterial infection, and improves tissue repair through photobiomodulation mechanisms​​. When comparing both modalities, each has shown unique advantages depending on the specific clinical application and parameters used. There is also evidence suggesting that combining PEMF with laser therapy could provide synergistic effects, further enhancing therapeutic outcomes in terms of pain relief and tissue healing^26^.

## Long term effect of PEMF therapy

The integration of long-term pulsed electromagnetic field (PEMF) therapy into medical treatments has shown a wide range of effects on different physiological systems. In animal studies, long-term exposure to PEMF has been observed to cause oxidative stress in hepatic and immune functions, notably increasing serum alanine aminotransferase and aspartate aminotransferase activities, leading to oxidative damage in the liver and spleen^27^.In another study, PEMF was shown to enhance cortical plasticity in the healthy human brain by modulating corticospinal excitability, providing potential neuroprotective benefits and opening new avenues for treating neurological disorders^28^. Additionally, research has indicated that PEMF can influence immune functions, such as reducing the secretion of immunoglobulins and weakening humoral immunity, which highlights its extensive impact on cellular activities and overall health^29^. Collectively, these findings underscore the broad applications and significant influence of long-term PEMF therapy in both experimental and clinical settings, emphasizing its therapeutic potential and the need for further research to optimize treatment protocols for various health conditions.

## Dental applications

In dentistry, PEMFs has shown a great impact, mostly in dental implantology. Dental implants are considered as an ideal treatment for oral rehabilitation of edentulous subjects, primary implant stability considered as a key factor influencing the final osseointegration outcome^30^. Therefore, there is a numerous necessity for supplementary treatment to overcome poor bone quality issues. Targeting the promotion of osteogenesis, consequently minimizing the required loading time^28^.

PEMFs stimulate bone formation, bone ingrowth of dental implants thus helping to decrease time for osseointegration that positively affect the time required for loading^31^. PEMFs enhance osseointegration through induction of osteoid formation and neovascularization^31^. PEMFs also induce bone repair processes by increasing expression of bone morphogenetic proteins 2 and 4. PEMFs promotes diﬀerentiation of osteoblast cells, and induction of osteogenesis^2^ and on the other hand reduce the activity of osteoclastic cells^2^. 

**Figure 2:**
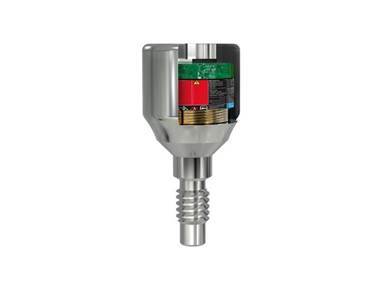
A cross-sectional view of the Miniaturized Electromagnetic Device (MED) healing abutment (Courtesy of Magdent Ltd.).

Nayak et al. developed PEMFs using a newly designed Miniaturized Electromagnetic Device (MED) (Magdent Ltd., Bnei Brak, Israel)^31^. This device, constructed from Ti-6Al-4V, incorporates a battery and a coil that follows the design of traditional healing abutments and is screwed into the implant (Figure 2). To initiate the electromagnetic field generation by the MED, an activator is required. Research participants were assigned to either a PEMF group, which received the MED, or a control group that was given a placebo-healing cap post-implantation. The study assessed implant stability through implant stability quotient (ISQ) measurements, resonance frequency analysis (RFA), radiological evaluations, and analysis of proinflammatory cytokines in peri-implant cervical fluid (PICF). RFA assessments were conducted immediately after implantation and then at intervals of 2, 4, 6, 8, and 12 weeks, while radiographic evaluations were carried out at the start, and then at 6 and 12 weeks^31^.

The findings of the study indicate that PEMFs significantly improve implant stability, particularly in the initial phases of healing, and enhance bone quality and development surrounding the implant when compared to conventional healing abutments. Moreover, a decrease in TNF-α levels was observed within the first four weeks after implantation in those receiving the MED healing abutments versus standard ones^31^. The conclusion drawn is that the MED contributes to enhanced stability of the implant at early stages.

**Figure 3:**
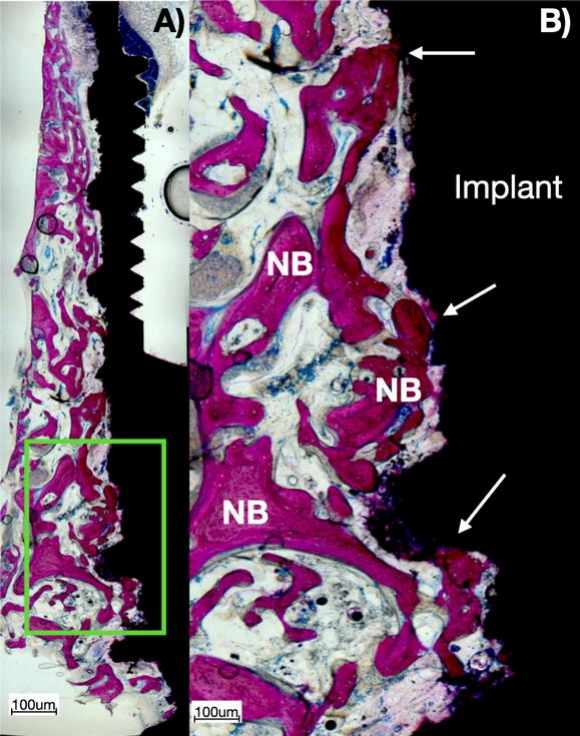
Histological ground section of experimental dental implant retrieved after 60 days of human maxilla with the use of MED applying PEMF: A) higher view of the section of the implant showing the bone-to-implant contact along the entire height of the implant; B) close view of the green square presented in A). Note that there was a direct ossification (arrows) after the effect of PEMF stimulating bone formation, ingrowth on dental implants, and increased bone stock, especially in type IV bone.

Histologically, the PEMF increases the direct ossification around dental implants placed in alveolar bone type IV or systemically compromised subjects with osteoporosis, diabetes, and smokers. Figure 3 shows a ground section of a retrieved mini-implant from the human maxilla after 60-day healing period.

Peri-implantitis is a common biological complication that may cause implant loss if untreated^32^. A variety of reasons has been linked to the initiation and progression of peri-implant illness, including an excess of cement, malpositioning, misfitting of the implant or abutment, and incongruences in the prosthesis^33^.

This issue can be addressed through both surgical and nonsurgical approaches^34^. Surgical solutions encompass open flap debridement (OFD) along with cleaning the surface of the implant, which can be performed with or without bone resection or regeneration. However, the predictability of these methods has yet to be fully established. On the nonsurgical side, treatments involve the use of ultrasonic devices, the localized application of antibacterial agents, lasers of various wavelengths, and manual or mechanical cleaning of the implant surface. These nonsurgical interventions have been shown to have a moderate effect^16^ ^,^ ^34^.

Previous study examined the use of MED on implants diagnosed with peri-implantitis^35^. After nonsurgical mechanical implant surface debridement was executed on all peri-implantitis affected implants. Participants were allocated to test of the control group. In the test group, MED abutments were connected while the control group received standard healing abutments. Pocket probing depth (PPD) and bleeding on probing (BOP), radiological (crestal bone loss) and immunological (crevicular fluid interleukin-1β levels) were collected.

Significantly less crestal bone loss was found in the test group after 1 and 3 months. Lower levels of the pro-inflammatory IL-1β levels were found in the test group for 2 weeks. In addition, improvement of the clinical parameters was found in the test group compared to the control group^35^.

## Current Challenges and Perspectives

While in vitro and in vivo studies have underscored the therapeutic potential of Pulsed Electromagnetic Fields (PEMFs), several critical challenges must be addressed to facilitate their translation into clinical practice.

### Limitations of In-Vitro Studies

In vitro studies, though informative, lack the complexity of living organisms. They do not fully capture the dynamic interactions present in human tissues, potentially leading to an overestimation of the efficacy and safety of PEMFs.

### Limitations of In-Vivo Studies

In vivo studies provide more comprehensive insights but often utilize animal models that may not accurately reflect human physiology and disease states. Variations in anatomy, metabolism, and genetic background can result in divergent treatment outcomes. Additionally, ethical and regulatory constraints may limit the scope of these studies, introducing potential biases.

Successfully translating preclinical findings into clinical practice requires addressing several hurdles. Standardization of PEMF parameters (such as frequency, intensity, and duration) is essential to ensure consistent outcomes. Moreover, large-scale, randomized controlled trials are necessary to establish the efficacy and safety of PEMFs across diverse patient populations and to account for potential confounding factors such as comorbidities and concurrent treatments.

To address these challenges, the use of advanced in vitro models (such as 3D cultures and organ-on-a-chip systems) can provide more physiologically relevant data. Enhancing the rigor of in vivo studies through improved animal models that better simulate human conditions can bridge the gap between preclinical and clinical research. Collaborative efforts among researchers, clinicians, and regulatory bodies are crucial to develop standardized guidelines and protocols for PEMF therapy.

## Conclusion

The manuscript highlights the evolution and therapeutic potential of Pulsed Electromagnetic Fields (PEMFs) across medical and dental fields, emphasizing their role in enhancing tissue healing and regeneration. It demonstrates the efficacy of PEMFs in improving bone healing, pain relief, and inflammation reduction. The introduction of the Miniaturized Electromagnetic Device (MED) marks a significant advancement, particularly in dental implantology, by promoting implant stability, osseointegration and antimicrobial effects. Overall, the study showcases the promising applications of PEMFs in advancing patient care and treatment methodologies.
